# Candidate Polyurethanes Based on Castor Oil (*Ricinus communis*), with Polycaprolactone Diol and Chitosan Additions, for Use in Biomedical Applications

**DOI:** 10.3390/molecules24020237

**Published:** 2019-01-10

**Authors:** Yomaira L. Uscátegui, Luis E. Díaz, José A. Gómez-Tejedor, Ana Vallés-Lluch, Guillermo Vilariño-Feltrer, María A. Serrano, Manuel F. Valero

**Affiliations:** 1 Doctoral Program of Biosciences, Universidad de La Sabana, Chía 140013, Colombia; yomairausma@unisabana.edu.co; 2 Energy, Materials and Environment Group, Faculty of Engineering, Universidad de La Sabana, Chía 140013, Colombia; 3 Bioprospecting Research Group, Faculty of Engineering, Universidad de La Sabana, Chía 140013, Colombia; luis.diaz1@unisabana.edu.co; 4 Centre for Biomaterials and Tissue Engineering, Universitat Politècnica de València, Camino de Vera, s/n, 46022 Valencia, Spain; jogomez@fis.upv.es (J.A.G.-T.); avalles@ter.upv.es (A.V.-L.); guivifel@upv.es (G.V.-F.); mserranj@fis.upv.es (M.A.S.); 5 Biomedical Research Networking Center in Bioengineering, Biomaterials, and Nanomedicine (CIBER-BBN), 46022 Valencia, Spain

**Keywords:** castor oil, biomedical devices, polyurethanes, polycaprolactone-diol, chitosan

## Abstract

Polyurethanes are widely used in the development of medical devices due to their biocompatibility, degradability, non-toxicity and chemical versatility. Polyurethanes were obtained from polyols derived from castor oil, and isophorone diisocyanate, with the incorporation of polycaprolactone-diol (15% *w*/*w*) and chitosan (3% *w*/*w*). The objective of this research was to evaluate the effect of the type of polyol and the incorporation of polycaprolactone-diol and chitosan on the mechanical and biological properties of the polyurethanes to identify the optimal ones for applications such as wound dressings or tissue engineering. Polyurethanes were characterized by stress-strain, contact angle by sessile drop method, thermogravimetric analysis, differential scanning calorimetry, water uptake and in vitro degradation by enzymatic processes. In vitro biological properties were evaluated by a 24 h cytotoxicity test using the colorimetric assay MTT and the LIVE/DEAD kit with cell line L-929 (mouse embryonic fibroblasts). In vitro evaluation of the possible inflammatory effect of polyurethane-based materials was evaluated by means of the expression of anti-inflammatory and proinflammatory cytokines expressed in a cellular model such as THP-1 cells by means of the MILLIPLEX^®^ MAP kit. The modification of polyols derived from castor oil increases the mechanical properties of interest for a wide range of applications. The polyurethanes evaluated did not generate a cytotoxic effect on the evaluated cell line. The assessed polyurethanes are suggested as possible candidate biomaterials for wound dressings due to their improved mechanical properties and biocompatibility.

## 1. Introduction

Polyurethanes (PUs) are widely used in the preparation of medical devices due to their biocompatibility, degradability, and non-toxicity when compared to polymers such as polylactic acid (PLA), polycarbonate, polycaprolactone, among others [1,2,3,4]. Examples of PU applications in the biomedical field are implants, artificial heart valves, sutures, catheters, artificial heart, vascular prostheses, wound coatings, blood compatible coatings, drug delivery systems, porous supports for tissue regeneration, among others [5,6,7,8,9].

Since the mechanical, thermal, chemical and biological properties of PUs can be varied during the synthesis process [10,11,12,13,14], the addition of polymers, such as polycaprolactone diol (PCL) or chitosan (Ch) can modify the properties of PUs such as biocompatibility [15] and the antimicrobial activity. PCL is an attractive polymer for the development of biomaterials due to its properties such as biocompatibility, biodegradability, ease in the processing of biomaterials, among others [16]. Ch is a polysaccharide that is obtained from renewable sources because it is part of the structure of some crustaceans. Ch is mainly characterized by being biocompatible, biodegradable, bioadhesive, non-the toxic, and has antimicrobial properties, among others [15,17,18]. These properties have allowed PCL and Ch to be used in some applications such as wound dressings, surgical sutures, scaffolds in tissue engineering, among others [19]. The use of Ch and PCL is expected to increase the biocompatibility of the PUs synthesized with polyols derived from castor oil. Additionally, the filler effect is expected to increase the mechanical properties such as tensile strength. And when using the mixture of chitosan with polycaprolactone, it is sought to evaluate if there is a possible synergistic effect or not to obtain biocompatible materials with antimicrobial properties, or if both mechanical and biological properties are affected.

Biocompatibility is interpreted as a series of interactions that occur at the tissue/material interface, allowing the identification of those materials with surface characteristics and/or more biocompatible polymer chemistry; these interactions are influenced by the intrinsic characteristics of the material. Some biocompatibility tests involve analytical tests or observations of physiological phenomena, reactions or surface properties attributable to a specific application [12].

Cell cultures are ideal systems for the study and observation of a specific cell type under specific conditions since these systems do not have the complexity that an in vivo system entails, due to a large number of variables that interact. In vitro tests assess the morphology, cytotoxicity and secretory functions of different cell types. The tests can be by direct contact of the cells and the material or indirect, adding an extract of the material to the cell culture [20,21].

Monocytes and macrophages are part of the innate immune system because they are cells that are involved in inflammatory processes with the ability to synthesize and secrete pro and anti-inflammatory cytokines [22]. Cytokines correspond to a diverse group of extracellular, water-soluble proteins, which influence the production and activity of other cytokines by increasing (proinflammatory) or decreasing (anti-inflammatory) the inflammatory response [23].

The human monocyte cell line THP-1 is widely used in research thanks to the ability of monocytes to differentiate into macrophages [24]. THP-1 monocytes have a round shape in suspension; when they differentiate upon stimulation by phorbol 12-myristate-13-acetate (PMA), the cells adhere to the culture plates, gaining phenotypic and functional characteristics similar to primary human macrophages [25,26]. The immune response is assessed by measuring cytokines in the cell culture medium [22].

The inflammatory response of macrophages is activated by invading pathogens, particles, lipopolysaccharides (LPSs), and other stimuli [27]. LPSs are part of the outer membrane of Gram-negative bacteria and can cause tissue damage and the release of multiple pro-inflammatory cytokines [27]. Therefore, when an inflammatory response is induced, pro-inflammatory cytokines, such as interleukin-1 beta (IL-1β), tumor necrosis factor alpha (TNF-α), and interleukin-6 (IL-6), can be released. Likewise, anti-inflammatory cytokines, such as interleukin-10 (IL-10) [28], can be released. Biomaterials, such as high molecular weight polyethylene, can activate macrophages to secrete pro-inflammatory cytokines, including TNF-α and IL-1β, among others, as a response to material implantation [29].

PU applications in biomedicine are diverse due to Pus’ various properties. Using aliphatic chains derived from vegetable oils creates flexible PUs, and cyclic diisocyanates provide greater mechanical strength [30]. Therefore, it is necessary to specifically characterize each synthesized material to determine its functionality and suitability for biomedical devices. The aim of this research was to determine the physicochemical, mechanical, morphological, biodegradability, and in vitro biocompatibility characteristics of the PUs, in addition to the possible inflammatory effects of the materials synthesized with castor oil polyols, depending on segment structure and PU cross-linking density. In this study, different PUs were synthesized with castor oil (chemically modified or not) and isophorone diisocyanate (IPDI) by adding polycaprolactone diol (PCL) (15% *w*/*w*) and chitosan (Ch) (3% *w*/*w*). In vitro degradation was determined in acidic and basic media, and enzymatic degradation was carried out with pig liver esterase. The in vitro cell viability was determined using L929 mouse fibroblasts (ATCC^®^ CCL-1), human fibroblasts (MRC-5) (ATCC^®^ CCL-171™), and adult human dermal fibroblasts (HDFa) (ATCC^®^ PCS-201-012™) with the PUs. The viability was also determined by a live/dead kit for the L929 mouse fibroblasts. Pro- and anti-inflammatory responses were evaluated by cytokine expression (IFN-γ, IL-1β, IL-2, IL-4, IL-5, IL-6, IL-8, IL-10, and TNF-α) of the THP-1 cells with and without stimulation by LPS. The present paper serves as a screening of the immunomodulatory effects of PU materials synthesized with castor oil.

## 2. Results and Discussion

### 2.1. Obtaining Polyols

**Scheme 1:**
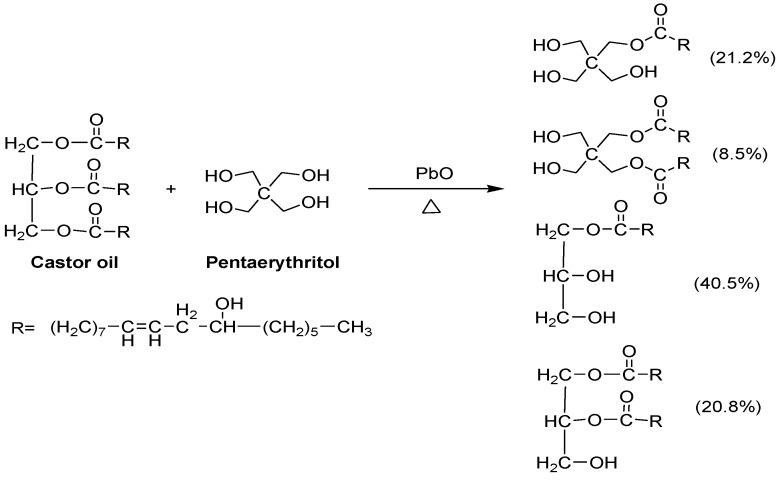
Reaction scheme for obtaining polyols.

The reaction to obtaining polyols derived from the castor oil is presented in Scheme 1. The hydroxyl number of the polyols transesterified with pentaerythritol was determined. The values of the hydroxyl index for each polyol (P.1, P.2 and P.3) were 160, 191 and 236 mg KOH per g of castor oil sample, respectively. According to the results of the hydroxyl index, it is noted that the chemical modification of the castor oil increases as the pentaerythritol content increases. The increment of the hydroxyl index can be related to the increase of the crosslinking reactions and to the gain of the bulk density of the polymeric materials. An increase in the crosslink density would generate an improvement in the mechanical properties of the polymer matrices.

### 2.2. Mechanical Properties of PUs

Aromatic diisocyanates are the most commonly used in the synthesis of PUs due to their mechanical properties, but they can produce carcinogenic and mutagenic diamines upon degradation [31], therefore, an aliphatic diisocyanate was used in this research to avoid the side effects of the raw materials on living tissues.

**Figure 1:**
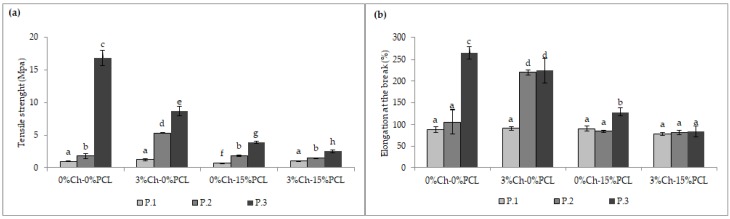
Mechanical properties of the synthesized PUs. (**a**) Maximum stress of PUs; (**b**) Percent elongation of PUs. The data are expressed as the mean ± SD (n = 3). Bars with different letters (a–h) indicate significant differences (*p* < 0.05) between the polyols.

Mechanical testing is essential to establish the use of a biomaterial because it allows obtaining the load parameters required for a tissue of interest [32]. The mechanical properties of 12 PU matrices were evaluated by determining the stress-strain curves from which the tensile strength and the elongation at the break were obtained. Figure 1 shows the results of the mechanical properties depending on the polyol used in the synthesis.

Physical and mechanical properties depend on the atomic and molecular structure of the materials used in the synthesis. The nature of the bonds and subunits of the structure affects the mechanical properties and therefore the stress-strain properties, which are of interest for the evaluation of biomaterials [32]. The highest tensile strength (16.86 MPa) was obtained with polyol P.3 (P.3-0%Ch-0%PCL). Figure 1a shows an increase in the tensile strength of all the materials synthesized with polyol P.3, which has the highest cross-linking. This is how the chemical modification of the polyols increases the maximum stress—via the increase of physical cross-linking of the polymer matrix [33].

The mechanical properties of PUs are attributed to presence of hard and soft segment domains [34]. The hard segment generally refers to the combination of the chain extender and the diisocyanate components, while the soft segments refer to polymeric diols. Depending on the structure of the hard and soft segments, crystalline and amorphous domains can be formed, which determine the stiffness and stability of the material [35]. Hydrogen bond cause strong interactions, so the polar nature of the hard segment causes a strong attraction, forming the domains [36]. Therefore, when using polyol P.3 in the synthesis, the values of the mechanical properties increased because the soft segments had a higher number of hydroxyl groups, increasing the cross-linking density, and the hard segments formed a ring, providing greater resistance.

Regarding the percent elongation at break (Figure 1b), the analysis showed significant differences (*p* < 0.05) of polyol P.1 compared with the other polyols (P.2 and P.3), with the percentage increasing as the polyol was modified. The mechanical properties of PUs depend on many factors, including molecular weight, chemical bonds, cross-linking, crystallinity of the polymer, and the size, shape, and interactions of the hard segment present in the structure [37]. Thus, PUs with a higher degree of cross-linking have higher values of tensile strength and percent elongation at break. Increased cross-linking produces a more compact structure [37]. An increase in strength is attributed to the content of intermolecular hydrogen bonds and cross-linking density [38].

When analyzing the influence of the additives used in the synthesis on the mechanical properties, significant differences are found when 3% Ch was added to polyol P.2 (P.2-3%Ch-0%PCL), obtaining a higher value than the other materials synthesized with P.1 and P.3. For polyol P.3, the additives decreased the maximum stress compared with the material without additives.

Chen et al. (2018) synthesized PUs with PCL as a polyol, IPDI, and polylactic acid (PLA), obtaining tensile strength values between 41 and 60 MPa when using PLA- and PCL-based PU ratios in the range of 80/20 to 95/5. The authors attributed the decrease in tensile strength as the ratio of PCL increased to the plasticizing effect of certain non-cross-linked PCL polyols and to a possible decrease in compatibility as the PCL content increased [39].

A similar effect was observed for percent elongation at break because it decreased when PCL and Ch were added to polyol P.3 (P.3-3%Ch-15%PCL). The other materials did not differ with the additives used from the material without additives. The flexibility of PU may be due to the long oil hydrocarbon chain present in the polymer chain [37]. This agrees with the results reported by Park et al. (2013), who synthesized PUs with polycaprolactone, hexamethylene diisocyanate, and isosorbide, with silk added, and determined that a higher silk content increased the stiffness and decreased the maximum stress. The authors stated that the design of flexible and soft polymers allows for the production of a wide range of biomaterials to regenerate soft tissues such as muscles and ligaments [6]. Additionally, Vannozzi et al. reported that in general, soft and deformable substrates are key features for skeletal muscle tissue engineering [33].

### 2.3. Fourier-Transform Infrared Spectroscopy (FTIR)

**Figure 2:**
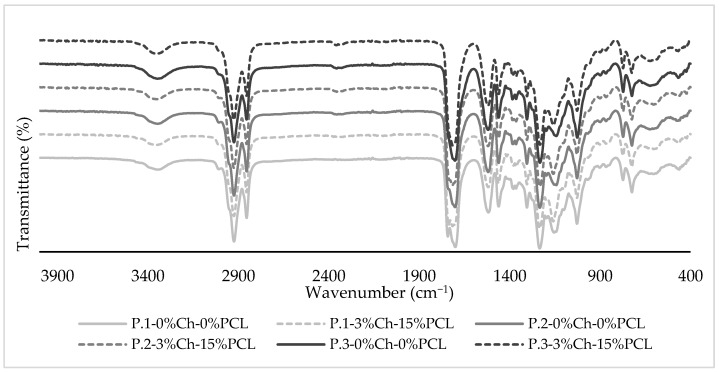
FTIR spectra of the synthesized PUs.

FTIR was used to determine the efficiency of the synthesis process by the identification of characteristic functional groups of PU and the absence of characteristic peaks of the monomers used in the synthesis process. Figure 2 shows the infrared spectra of the synthesized PUs.

Figure 2 shows that all of the FTIR spectra had similar peaks, independent of the polyol or additive used in the synthesis, and the peaks observed corresponded to the expected PU matrices. The absence of the stretching peak of the −N=C=O bond of the diisocyanates at 2250 cm^−1^ [31], indicates there were no unreacted free isocyanate groups in the synthesized PU matrices, showing that the reaction was complete.

Spectral peaks characteristic of PUs can be seen in the spectra. Thus, around 3330 cm^−1^, the characteristic bands for the stretching vibrations of the −N–H bonds are observed [40] from which it can be inferred that they correspond to the urethane bonds present in the matrices. Near 2923 cm^−1^, the stretching peak of the methyl group can be observed, and at around 2855 cm^−1^, the symmetric stretching of the C–H bond is present. At around 1700 cm^−1^, an intense band is present due to the C=O bond stretching [40] thus indicating the formation of the urethane group. Around 1250 cm^−1^, the C–N bond stretching is observed; at about 1140 cm^−1^, the stretching vibrations of the C–O bond appear [37].

### 2.4. Thermal Analysis

#### 2.4.1. Thermogravimetric Analysis

**Figure 3:**
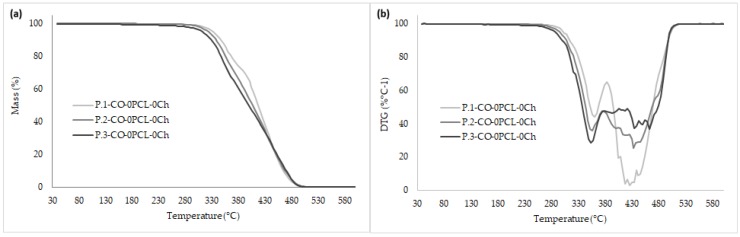
Thermograms of the synthesized PUs. (**a**) Thermogravimetric (TG) curve of PUs; (**b**) Derivative of the thermogravimetric (DTG) curve of PUs.

Thermogravimetric analysis was performed by producing thermograms of the PUs. Figure 3 shows the results for the synthesized matrices.

Figure 3 shows a trend regarding thermal behavior of the synthesized PUs, showing no displacements of the degradation temperatures with the use of modified polyols. When obtaining the curves derived from thermogravimetry, several peaks were observed, which agrees with other authors stating that the mechanism of degradation of PUs is complex due to the formation of various compounds in the process [41].

For each polymer matrix a thermogram was obtained from which the stability of the synthesized PUs in this study were determined. It was observed that the polyol type and additives did not affect the thermal stability of the materials when compared with the synthesized material without additives. The thermograms showed that all PUs were stable at temperatures below 300 °C and showed complete degradation at temperatures near 600 °C, which coincides with the research conducted by Jutrzenka et al. (2018), who synthesized PUs based on a glycerin derivative, polyethylene-butylene, and diphenylmethane diisocyanate. They determined that the materials were stable up to 300 °C [36]. In a study on PUs synthesized with castor oil and isophorone diisocyanate that were proposed as surgical adhesives, the PUs had the same degradation values, and the authors stated that the values related to the degradation temperatures do not affect the biomedical application since physiological temperature is lower (≈37 °C) [42].

Three degradation regions were detected for the polymer matrices. This is consistent with the study performed with polymeric matrices synthesized with castor oil and isophorone diisocyanate by adding different concentrations of PCL and Ch [43]. The first stage of degradation was observed in the range of 250–370 °C and corresponds to the thermal degradation of the urethane bonds formed in the hard segments, characterized by being technically unstable [2]. The second stage was between 375–430 °C and corresponds to the degradation of the soft segments [36]. The last stage was in the range of 425–500 °C and corresponds to the thermal degradation of the double bonds of remaining fatty acids from castor oil [44,45].

#### 2.4.2. Differential Scanning Calorimetry (DSC)

Table 1 shows the glass transition temperature (*T_g_*) values for the synthesized materials, as determined by the DSC curves of the materials. Table 1 shows that the polyol type and the additives significantly influence the *T_g_* value. This agrees with what is understood from the mechanical properties of the molecular structure produced by modifying the polyol derived from castor oil. The values of *T_g_* for P.1, P.2, and P.3 without additives were −14.8 °C, −12.8 °C and 14 °C, respectively. P.1 had the lowest values of *T_g_* (between −14 °C and −25 °C, approximately).

The observed trend is a decrease in *T_g_* as the hydroxyl index decreases, which agrees with the cross-linking of the synthesized PUs, indicating that greater energy is needed for reordering the structure by a change in intermolecular forces. This may be due to secondary interactions resulting from the hyperbranched structure [46]. The thermal properties of PUs depend on the number of urethane bonds present in the structure because they can tolerate a considerable amount of heat [47]. Saénz-Pérez et al. (2016) synthesized PUs with polytetramethylene glycol and diphenylmethane or toluene diisocyanates, and butanediol. In the determination of *T_g_*, they found that the values increased as the amount of chain extender increased. Therefore, the authors state that the increase in *T_g_* was caused by the reduction in the mobility of chain segments due to the increase of hard segments [48].

The ricinoleic acid triglyceride from castor oil used as a polyol contains an ordered structure in which hydroxyl groups are uniformly distributed within the chain, helping to obtain a PU with a uniform cross-linked structure, achieving high mechanical properties and thermal stability [30]. In general, all of the synthesized matrices had a single value of *T_g_*, indicating that all the materials showed homogeneous segment dispersion. The *T_g_* values were similar to those reported for PUs based on polyethylene glycol, poly (ɛ-caprolactone-co-D,L-lactide), and diurethane diisocyanate (with hexamethylene diisocyanate and butanediol), where the authors found values near −33 °C. Likewise, the authors did not find exothermic peaks because the materials were amorphous [49]. With the above information, it can be generalized that Pus are thermally stable and that they can be used in various biomedical applications, for example, as materials for non-absorbable sutures.

### 2.5. Hydrophilic Character

#### 2.5.1. Contact Angle

**Figure 4:**
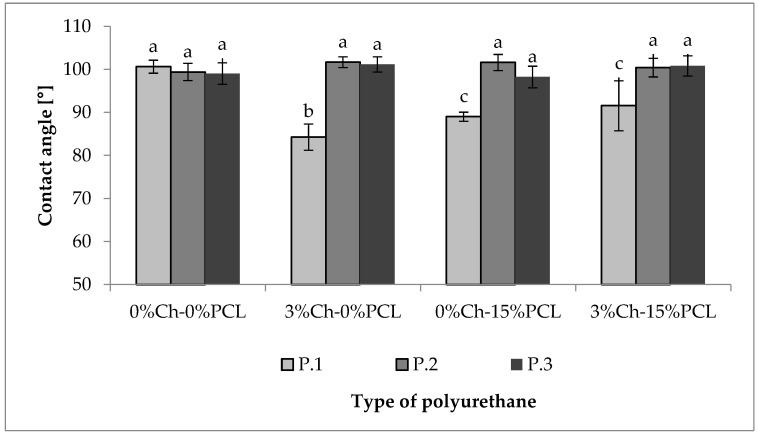
Contact angle of the synthesized PUs. The data are expressed as the mean ± SD (n = 10). Bars with different letters (a–c) indicate significant differences (*p* < 0.05).

To evaluate the hydrophilic nature of PUs, the water contact angle on their surfaces was determined. Figure 4 shows the results of the synthesized PUs.

According to the statistical analysis in Figure 4, it is observed that for polyols P.2 and P.3, there are no significant differences in contact angle values. For P.1, significant differences are observed by using additives because there is a reduction of angle values, indicating a decrease in the hydrophilic character. Values near 100 degrees were found, so it can be deduced that the materials tend to be hydrophobic. Mi et al. determined the contact angles of PCL-based thermoplastic PUs and different chain extenders; the values were near 90 degrees. They attributed the results of the contact angle to the hydrophilic functional groups present in the chain extenders that were used. The authors state that although PUs maintain the same chemical structure, monomer variation in the synthesis can affect the wettability and therefore vary the degradation behavior [50].

Likewise, Gossart et al. evaluated the contact angle of PUs synthesized with l-lysine diisocyanate, hydroxyethyl methacrylate, and poly (hexamethylene-carbamate) and found values above 80 degrees. The authors state that the common values reported for PU matrices were in the range of 80 to 90 degrees depending on the structure of the PU and interactions with the surfaces [51]. The results of the contact angle shown in Figure 4 are greater than those reported; this possibly results from the cross-linked network generated by the monomers used in the synthesis, as the materials tended to be hydrophobic.

#### 2.5.2. Water Absorption Rate

**Figure 5:**
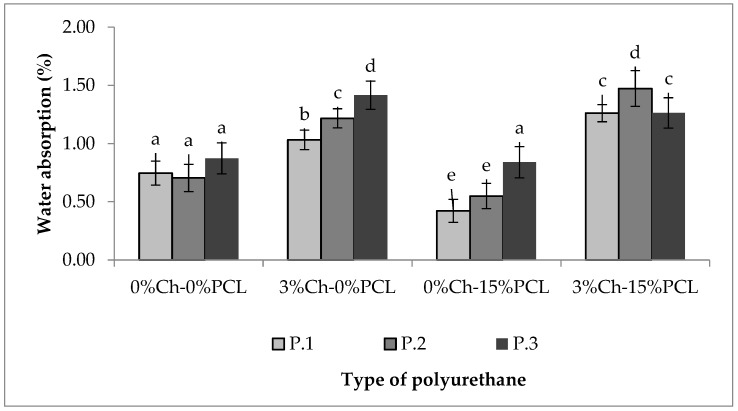
Water absorption rate over 72 h. Absorption results are expressed as the mean ± SD (n = 3). Bars with different letters (a–d) indicate significant differences (*p* < 0.05).

Figure 5 shows the rates of water absorption for the synthesized PUs during 72 h of testing.

The weights of the materials were monitored until constant weight over 24, 48, 72, and 144 h, but the data are not shown because no significant differences were found after 48 h. The results show that the water absorption rates range between 0.5 and 1.5%. As seen in Figure 5, adding PCL and Ch increases the rate of absorption compared to the material without additives, although the difference in rates was not greater than one. It is likely that by increasing the amount of additive more functional groups became available to interact with the medium, which is also polar. However, by increasing the functionality of the polyol, the effect generated is inverse, showing a reduction in the rate of absorption compared to PUs without the additive. Internal interactions (hydrogen bonds) increase the barrier effect by preventing fluid diffusion. In addition, the additive function causes the chains to reorganize, showing a reduction of volumetric defects or voids in which the water can be deposited [32].

Marques et al. evaluated a bioadhesive synthesized from lactic acid in which the water absorption was 10%. The authors noted that moderate rates of water absorption improved the hemostatic character of the materials [52].

The contact angle and the rate of absorption provide information about hydrophobicity/hydrophilicity and could be an indirect indicator of surface molecular mobility. Surface wettability can affect protein adsorption on the surface and biocompatibility [53]. This is one reason why it is indispensable to perform a study of material biocompatibility to determine its possible acceptance by the human body. Therefore, the PUs synthesized in this study can be considered suitable for biomedical applications, such as materials for non-absorbable sutures, considering the water absorption rates under the test conditions.

### 2.6. Density Determination

**Figure 6:**
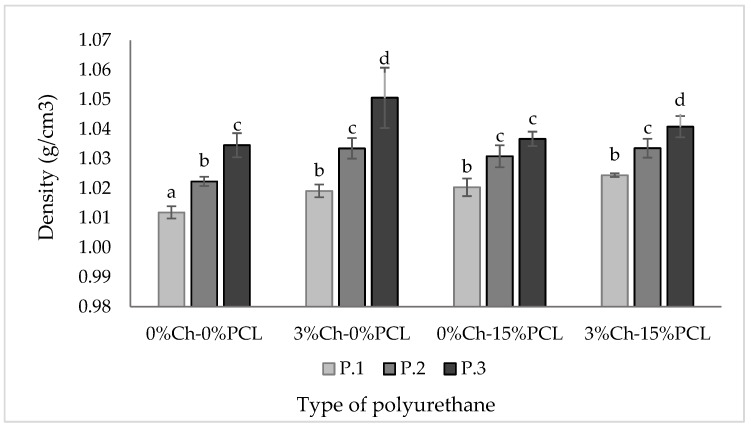
Density of the synthesized PUs. The data are expressed as the mean ± SD (n = 3). Bars with different letters (a–d) indicate significant differences (*p* < 0.05).

Figure 6 shows the density of the materials synthesized with IPDI and the additives (PCL and Ch).

As seen in Figure 6, the density of PUs depends on the type of polyol, which agrees with the results shown for the mechanical and thermal properties. As the cross-linking density increased, the density of the resulting polymeric material significantly increased, although the difference between the highest and lowest values was less than 5%. This agrees with the results of Conejero-García et al. who synthesized polyglycerol sebacate, with a different degree of cross-linking, as a material for various applications in tissue engineering. The density values reported by the authors ranged between 1.13 and 1.14 g mL^−1^ [54].

The high densities of polymeric materials can be related to a higher hydroxyl (OH) content due to increased cross-linking reactions [55]. In a study conducted by Carriço et al. they found that increasing the castor oil content in the formulation of foam increased the apparent density, suggesting that the polymer chains were more packed, with less free volume and smaller cells, increasing the stiffness of these materials [55].

### 2.7. Dynamo-Mechanical Thermal Analysis (DMTA)

**Figure 7:**
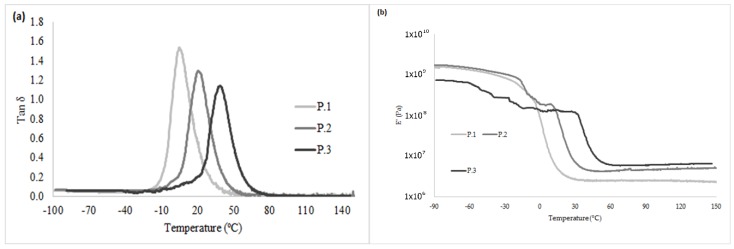
DMTA thermograms of the synthesized PUs. (**a**) Loss factor; (**b**) Storage modulus.

Figure 7 shows the dynamic behavior, DMTA, in a tension mode of PUs corresponding to the evolution of the modulus and the loss factor versus temperature. With the variation of the storage modulus and the loss factor, it was possible to observe the displacements suffered by *T_g_* for the evaluated materials. It can be seen that *T_g_* increases when polyols P.2 and P.3 are used; this is probably due to the stiffness of the structure and greater generation of hydrogen bonds because of the high hydroxyl index present in the polyol [38,39]. According to the above results, it can be inferred that material compatibility decreases when the polyol without modifications, and therefore with less cross-linking, is used.

Results with a similar trend were found by Chen et al. on PU matrices synthesized with PCL as a polyol, IPDI, and PLA. The authors reported that when the mobility of the chain decreased, the value of *T_g_* increased. Therefore, they observed that phase compatibility decreased when the PCL content increased due to a possible plasticizing effect of non-cross-linked material [39].

A similar behavior occurred with the modulus results. The lowest values corresponded to the materials synthesized with P.1 (polyol without modification) compared with polyols P.2 and P.3, which have a higher content of hydroxyl groups. An increase in hydroxyl groups results in an increase in cross-linking, hindering polymer chain mobility and thereby increasing the storage modulus. A decrease in polymer chain mobility can limit energy transfer and diffusion, which could decrease the absorption capacity of impact resistance and deformation [39].

The relationship between hard and soft segments is important as they simultaneously act as a physical cross-linking agent and as a high-modulus filler. When there is an organization of the hard and soft segments in the respective domains, pre-polymers tend to have two *T_g_* values. One temperature will be negative corresponding to the soft segments, while the other will be positive, corresponding to the hard segments [38]. When a single event of *T_g_* occurs, it could be inferred that there is a homogeneous phase distribution [38]. According to the results obtained by DSC, it can be observed that the trend of *T_g_* is similar, that is, a single value of *T_g_* is present, increasing as the polyol is modified. The presence of a single transition can be related to the existence of a dominant phase, so it can be inferred that there is a uniform distribution of the components [56]. The differences in *T_g_* between the polyols used may be due to the cross-linking density because this would cause a greater compatibility between hard and soft segments [38].

### 2.8. Field-Emission Scanning Electron Microscopy (FESEM)

**Figure 8:**
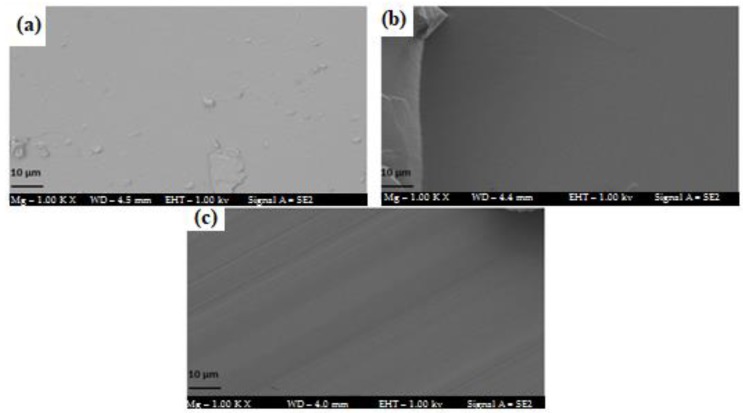
FESEM micrographs of PUs synthesized at 100×. (**a**) P.1; (**b**) P.2; (**c**) P.3.

Figure 8 shows the morphology of PUs synthesized with IPDI as a function of the polyol. The FESEM micrographs showed a uniform distribution of PUs, but it was not possible to differentiate the hard segments from the soft segments. Similarly, no differences were observed related to the type of polyol used for the polymeric matrix. These results can be correlated with the calorimetric results because if there is only a single *T_g_*, it is probable that there is a homogeneous phase distribution. This agrees with the results reported by Thakur et al. on PUs synthesized with castor oil and toluene diisocyanate for the coating of materials [47].

### 2.9. In Vitro Biodegradability Assays

**Figure 9:**
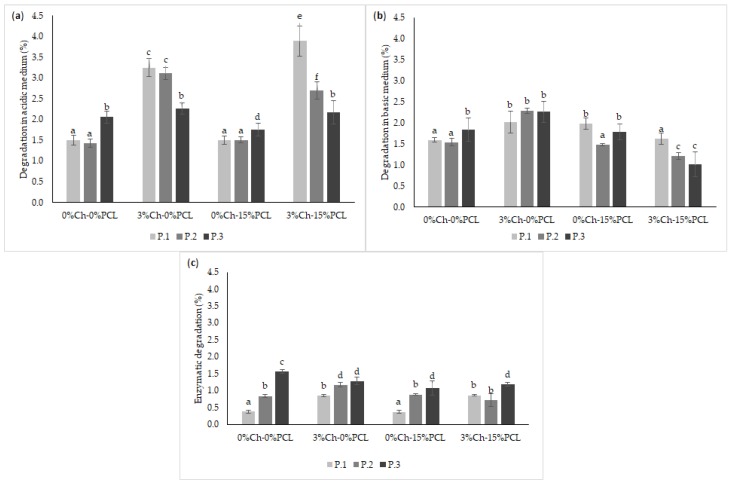
Degradation rate of PUs in different media. (**a**) Acidic medium (0.1 M HCl) for 90 days; (**b**) Basic medium (0.1 M NaOH) for 90 days; (**c**) Enzyme medium (esterase) for 21 days. The data are expressed as the mean ± SD (n = 3). Bars with different letters (a–f) indicate significant differences (*p* < 0.05) between the polyols.

The biodegradability of PUs was evaluated in the presence of different media (HCl, NaOH, and enzymatic) over a specific period of time, the results are shown in Figure 9.

PCL was used as a positive control in enzymatic degradation, showing a degradation of 9.08 ± 0.30% after 21 days. It can be inferred that the linear structure of PCL facilitates the diffusion of the cleaved chains throughout the polymer and their release into the medium, producing a higher degradation rate. For the synthesized PUs, characterized by being cross-linked materials, the chain mobility is lower, thus hindering the diffusion of the cleaved chains.

**Figure 10:**
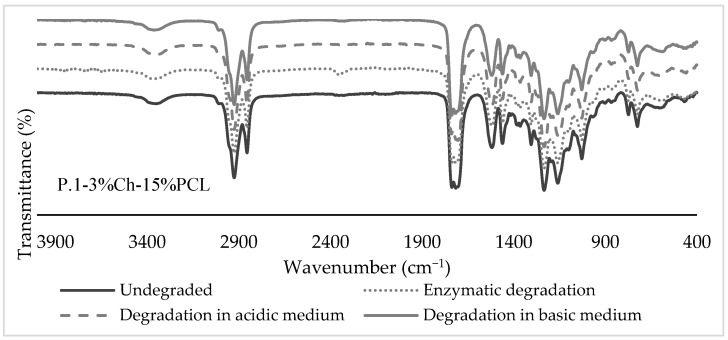
FTIR spectra of PUs degraded in acidic, basic, and enzymatic media.

The highest value of the degradation rate after 90 days of testing (acidic and basic media) was obtained with the acidic medium under the test conditions for the material synthesized with polyol P.1 by adding the additives (3%Ch-15%PCL). FTIR of the degraded materials (Figure 10) helped determine whether the degradation corresponded to one of the functional groups of the material.

**Figure 11:**
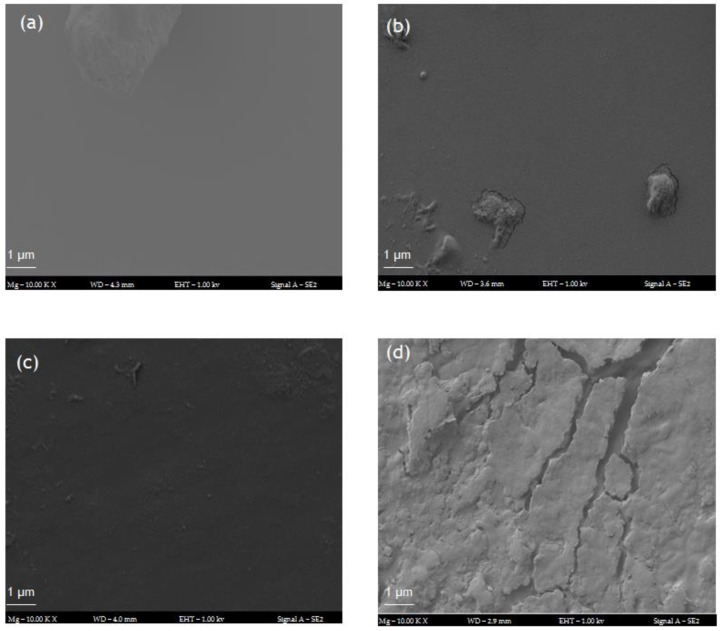
FESEM micrographs of the PU synthesized with P.2-3%Ch-15%PCL after the degradation process in different media (10,000×). (**a**) Undegraded material; (**b**) Acidic medium (0.1 M HCl) after 90 days; (**c**) Basic medium (0.1 M NaOH) after 90 days; (**d**) Enzyme medium (esterase) after 21 days.

Figure 10 shows that the functional groups characteristic of PUs are conserved compared with the undegraded material, therefore, it can be inferred that degradation occurs at the surface level. Surface images of the degraded materials were also taken using FESEM, observing a possible surface degradation of the materials (Figure 11). When observing that the degradation rate is less than 4% in the evaluated media, it can be said this is due to the hydrophobic character of PUs, which agrees with the results found for the rate of water absorption and contact angle.

The results obtained in this research show a similar trend with those reported by Thakur et al., who evaluated the chemical resistance of PUs in acidic and basic media, finding that in general, PUs show chemical resistance, and PUs based on castor oil showed higher resistance to the basic medium because the oil contains hydrolyzable functional groups [37]. This may be the reason why the biodegradation of the PUs in basic medium was lower than in acidic medium.

The degradation of poly (ester-urethane) occurs mainly by a hydrolytic attack of the ester and urethane bond [31]. Das et al. attributed the degradation in acidic and basic media to different types of strong interactions in the structures of PUs [57]. The authors stated that low resistance to alkalis is due to the hydrolyzable ester bonds in the monoglyceride residues and PCL of polymers [57]. After 90 days of testing, it was observed that the highest values of the degradation rate under the test conditions were relatively low (3.9% in acidic medium and 2.3% in basic medium). These results indicate that the polymeric materials are resistant to degradation due to the structure of PUs, that is, to the cross-linked matrix with high mechanical properties and a hydrophobic character.

**Figure 12:**
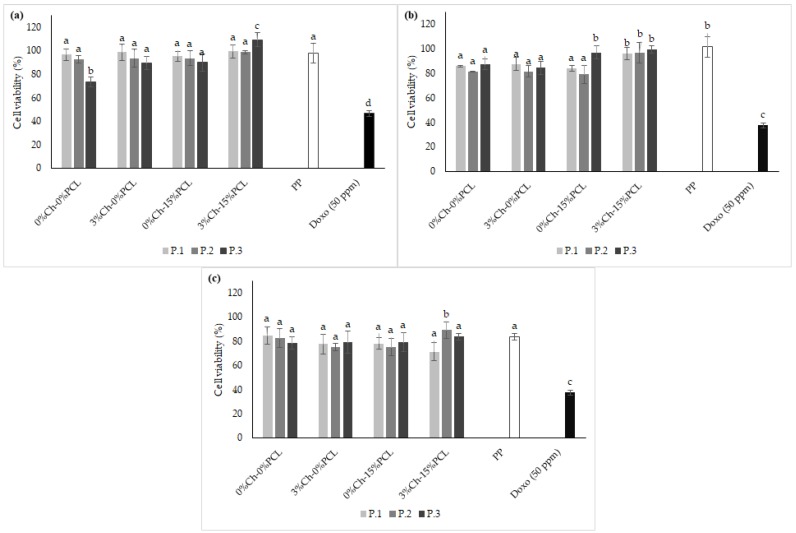
Percentage of cell viability at 24 h. (**a**) L929 Mouse fibroblasts; (**b**) Human fibroblasts (MRC-5); (**c**) Human dermal fibroblasts (HDFa). The data are expressed as the mean ± SD (n = 3). Bars with different letters (a–d) indicate significant differences (*p* < 0.05) between the polyols.

Regarding enzymatic degradation (Figure 12c), the highest rate observed was 1.6% after 21 days. This degradation process was greater than those obtained with the other treatments evaluated. This behavior can be attributed to an increase of hydroxyl groups in the polyols, producing an increase in the physical cross-linking of the polymer, and therefore, an increase in urethane groups. Urethane bonds are similar to amides and may be hydrolyzed by enzymes such as the esterase used in this research [58,59]. This agrees with what was stated by Gogoi et al. who reported that amide and urea bonds present in the branched polymer structure facilitate degradation [60]. According to Cherng et al., the degradation of PUs is due to cleavage of the hydrolytically weak bonds that are characteristic of the soft segments; therefore, they concluded that the in vitro degradation rate depends mainly on the type of polyol used in the synthesis due to the ester bonds in the structure [58].

### 2.10. In Vitro Cell Viability Assay by the MTT Method

As part of the in vitro biological evaluation of PUs, the percentage of cell viability was determined on three fibroblast cell lines. The results are shown in Figure 12.

Figure 12 shows that all polymers had a cell viability of greater than 70% for the three cell types evaluated. According to the ISO/CD 10993-5 standard, values greater than 70% can be considered suitable for biomaterials since they would be non-cytotoxic. As a control material, polypropylene (PP) was used because it is a biocompatible and non-absorbable biomaterial. As a negative control, doxorubicin (Doxo) was used. Figure 12a shows the cell viability results of the PUs on L929 fibroblasts. The percent viability of all matrices, except for the PU synthesized with P.3 without additive, did not show significant differences (*p* < 0.05) compared to PP. For the PU synthesized with P.3, significant differences were observed with the polymer synthesized with P.1 and with the control, but the cell viabilities are suitable to propose the synthesized materials as possible biomaterials. For the L929 fibroblasts, as the cross-linking of PU increased, the percentage of cell viability decreased. It is likely that the high degree of cross-linking produces a low availability of functional groups, such as −OH groups, in the cross-linked polymer. These groups would decrease the cell adhesion to the surface [33] mediated by proteins from the medium.

Bakhshi et al. evaluated PUs synthesized by adding quaternary ammonium salts in the epoxidation of soybean oil and found that PUs show a cell viability between 78–108% with L929 mouse fibroblasts, indicating that there was no toxicity of the synthesized polymers, coinciding with the results of this study [2].

Similar results were found by Calvo-Correas et al. in a preliminary study of in vitro cytotoxicity with murine L929 cells. These authors determined that PUs synthesized from castor oil and lysine diisocyanate had a cell viability greater than 100% in the first 24 h of the assay when following the ISO 10993-12 standard, indicating that the synthesized PUs were non-toxic and could have a potential use in biomedical applications [61]. Likewise, the results from the current study agree with those reported by Reddy et al., who synthesized a PU from lysine diisocyanate, PCL, and 1,4-butane-diamide and found that the polymers did not show toxicity when in contact with NIH/3T3 mouse fibroblasts [62].

Figure 12b shows the cell viability results of the polymers on human fibroblasts (MRC-5). For this cell line, no trend was observed related to the polyol type used in the synthesis of the PUs. The viability was decreased compared to that of the control material (PP); nevertheless, the materials are suitable for use in the design of biomaterials because they show a cell viability of greater than 80%.

Figure 12c shows the cell viability results of PUs on human fibroblasts (HDFa), and the materials show greater than 80% cell viability. Here, there were no statistically significant differences (*p* < 0.05) between the polyols used in the synthesis of PUs. Similarly, there were no significant differences between the synthesized materials and the PP control material; therefore, it can be inferred that the synthesized PUs can be considered suitable for the design of biomaterials for non-absorbable sutures.

The above results are in accordance with those reported by Coakley et al., who evaluated HDFa cells in vitro from a construct derived from porcine urinary bladder as a potential scaffold in tissue engineering. The study demonstrated the viability of the human dermal fibroblasts. The results showed cell viability values of greater than 90% [63].

Cytotoxicity may be related to the relative cell viability of controls, where values lower than 30% indicate severe toxicity of the materials, values between 30 and 60% indicate moderate toxicity, values between 60 and 90% indicate slight toxicity, and values above 90% indicate that the materials are non-toxic [64]. Therefore, the fibroblast cell viability results shown in Figure 12 demonstrate that the PUs are non-toxic for all of the lines evaluated.

### 2.11. Immunocytochemical Techniques

#### 2.11.1. In Vitro Cell Viability Assay by a Live/Dead Kit

**Figure 13:**
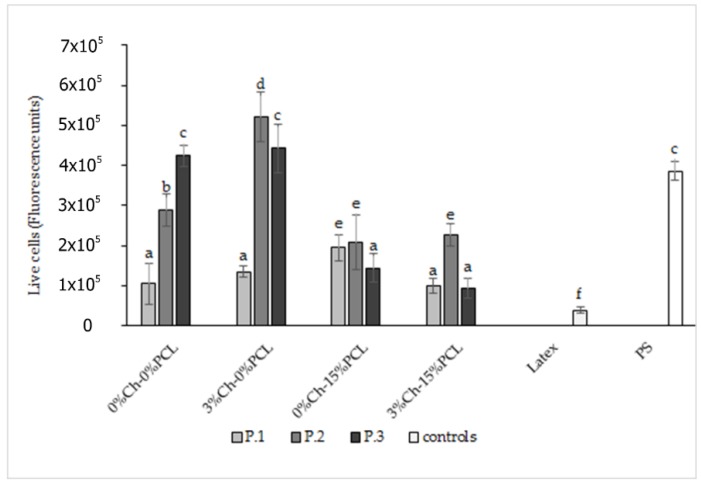
Fluorescence unit results of the in vitro cell viability test after 48 h with the live/dead kit. Polystyrene (PS) positive control; Latex negative control. The data are expressed as the mean ± SD (n = 3). Bars with different letters (a–f) indicate significant differences (*p* < 0.05) between polyols.

L929 cells that were cultured directly on the material and evaluated by the MTT method showed no toxic effects. When evaluating the cells by a live/dead viability kit, it was possible to observe that the cells adhered to the material, showed viability, and proliferated during the exposure time with the material. This kit allows determination of the number of live and dead cells during the test. In this experiment, viable cells were determined. Figure 13 shows the fluorescence units of the viability results as evaluated by the live/dead kit.

The results shown in Figure 13 indicate that PUs synthesized with modified polyols by transesterification with and without the addition of Ch show values of living cells similar to and higher than the material used as a positive control, PS. The materials synthesized with modified polyols by adding Ch showed statistically significant differences compared to the other PUs after 48 h of testing. Likewise, all the materials showed higher values than the negative control used in the test.

All the materials showed statistically significant differences compared to the negative control (latex). When comparing to the results of the positive control (PS), no significant differences were observed between the materials synthesized with P.3 without additive and by adding 3% Ch. This indicated that these materials had a similar cell viability behavior to that obtained with the PS reference material, which is widely used to grow and proliferate cells in vitro. Similarly, it was observed that one material evaluated showed significant differences compared to PS, with the viability values being higher than the positive control. This PU corresponds to the one synthesized with polyol P.2 with 3% Ch. These results indicate that Ch has a positive effect because it improves the biocompatibility of the PU.

Chitosan has a primary amino group and two free hydroxyl groups for each glucose unit, which is beneficial for biomedical applications [15]. The presence of free amino groups causes an increase in the positive charge of the polymer; therefore, there is a greater interaction between Ch and the cells [65].

These results agree with those reported by Laube et al., who performed a live/dead staining assay of NIH/3T3 fibroblasts grown on PU foams that were synthesized with lysine diisocyanate. They found that the cells were viable and proliferated, showing an increased cell density over time. Therefore, the authors proposed these materials as possible substitutes for soft tissue fillers [31].

#### 2.11.2. Fixation and Morphological Analysis with Phalloidin and DAPI

**Figure 14:**
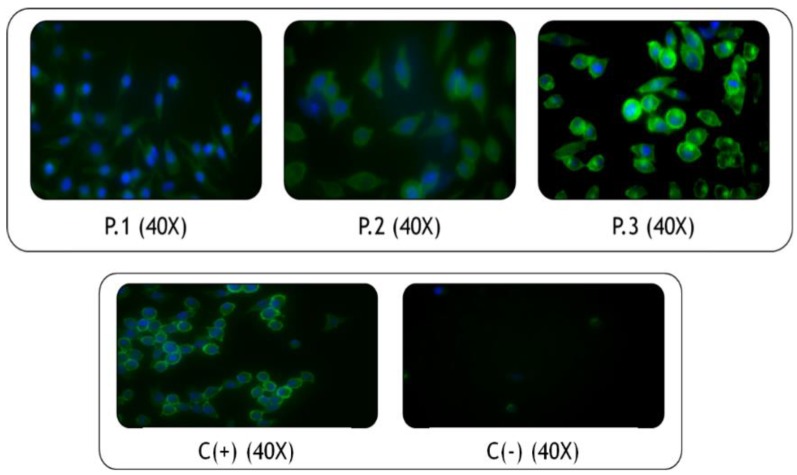
Optical microscopy images of phalloidin (green) and DAPI (blue) staining for representative PUs evaluated on L929 fibroblasts for 24 h. C(+) polystyrene positive control, C(−) latex negative control. Images were analyzed by ImageJ software.

In this assay, the adhesion and proliferation of L929 cells were observed by morphological analysis and were compared to the latex negative control. PS was used as a positive control, which is a common biomaterial used for cell culturing. Figure 14 shows the images obtained from the cells fixed and stained after 24 h of contact with the PUs by polyol type.

This technique stains the F-actin fibers (a main component of the cytoskeleton) with phalloidin, producing a green color [66], and DAPI staining produces a blue color for the fibroblast nuclei. As observed in the images, the structure of the L929 fibroblasts was conserved when compared with the positive control. Likewise, varying type of polyol or additive did not produce conformational changes in cell structure. As seen in the images, fibroblasts could adhere to the polymeric material, as evaluated after 24 h of contact. Cell morphology can indicate substrate-cell interactions. Flat and extended cell forms indicate strong adhesion from cell to substrate, while a rounded morphology generally means that the cells have difficulty in adhering to the substrate [67]. With the above, it can be inferred that the PUs evaluated in direct contact with cells allow cell proliferation and do not affect the cell morphology after 24 h of testing. Therefore, the synthesized PUs in this research may be suitable as materials for the design of non-absorbable sutures.

### 2.12. Evaluation of Inflammatory Processes

Twelve materials synthesized with castor oil polyols and IPDI with 3% Ch and 15% PCL were evaluated to determine the in vitro inflammation processes produced after contact with the materials. Inflammation was evaluated by the release of different cytokines to the medium of THP-1 cells that were differentiated into macrophages, with and without LPS stimulation, and in contact with the PUs. Nine inflammatory markers were analyzed, including pro- and anti-inflammatory cytokines (IFN-γ, IL-1β, IL-2, IL-4, IL-5, IL-6, IL-8, IL-10, and TNF-α). The first test group (group A) was used to determine the possible effect of PUs on the inflammatory process of macrophages.

**Figure 15:**
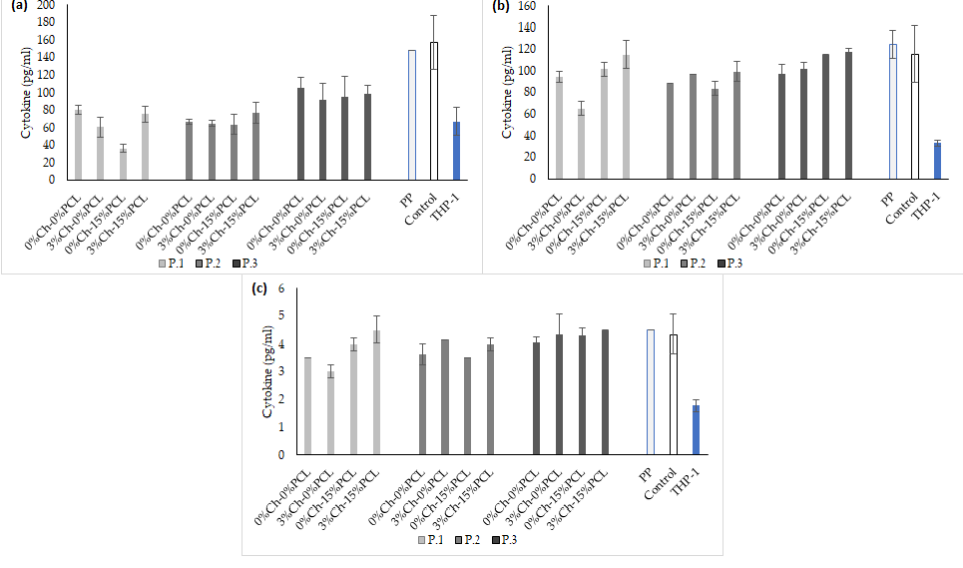
Anti-inflammatory cytokines without LPS stimulation. (**a**) IL-10; (**b**) IL-5; (**c**) IL-4. PP: polypropylene as a reference material; Control: culture medium without PUs; THP-1: monocyte cell line. The concentration of cytokines is expressed as the mean ± SD (n = 3).

**Figure 16:**
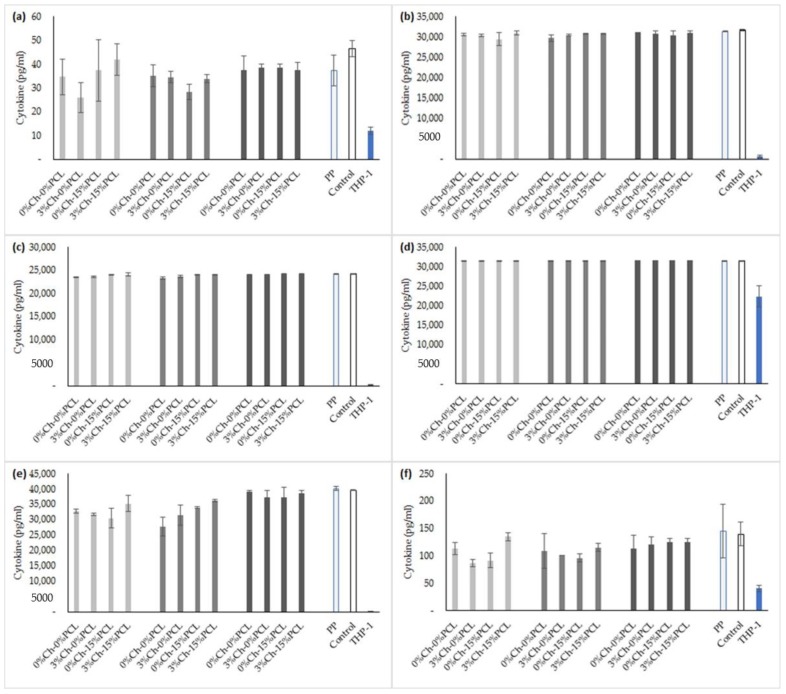
Pro-inflammatory cytokines without LPS stimulation. (**a**) INF-γ; (**b**) TNF-α; (**c**) IL-1β; (**d**) IL-8; (**e**) IL-6; (**f**) IL-2. PP: polypropylene as a reference material; Control: culture medium without PUs; THP-1: monocyte cell line. The concentration of cytokines is expressed as the mean ± SD (n = 3).

Figure 15 shows the concentrations of anti-inflammatory cytokines expressed by the macrophages after 24 h of exposure to the PUs. Figure 16 shows the concentration of pro-inflammatory cytokines expressed in the culture medium. The reference material was a polypropylene (PP) biomaterial used in non-absorbable biomedical sutures. The control was the culture medium without polymer. Figure 15 and Figure 16 show cytokine expression in the monocytes (the cells not treated with PMA). The second test group (group B) was formed to determine if the synthesized materials had anti-inflammatory activity against cells in which inflammation had been stimulated by LPS before direct contact with the PUs.

**Figure 17:**
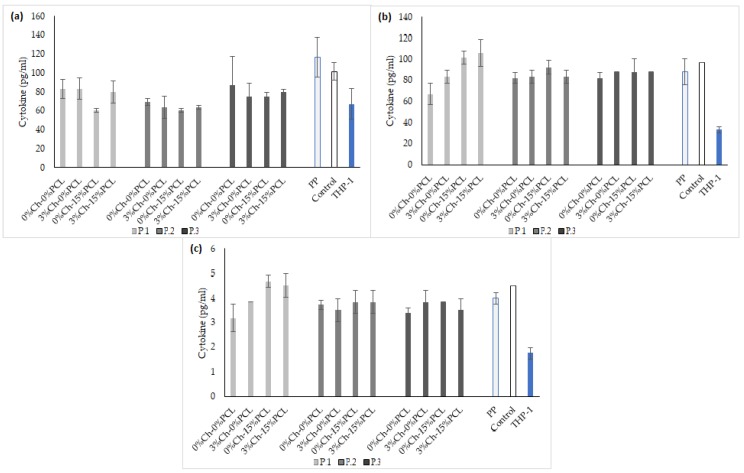
Anti-inflammatory cytokines with LPS stimulation. (**a**) IL-10; (**b**) IL-5; (**c**) IL-4. PP: polypropylene as a reference material; Control: culture medium without PUs; THP-1: monocyte cell line. The concentration of cytokines is expressed as the mean ± SD (n = 3).

**Figure 18:**
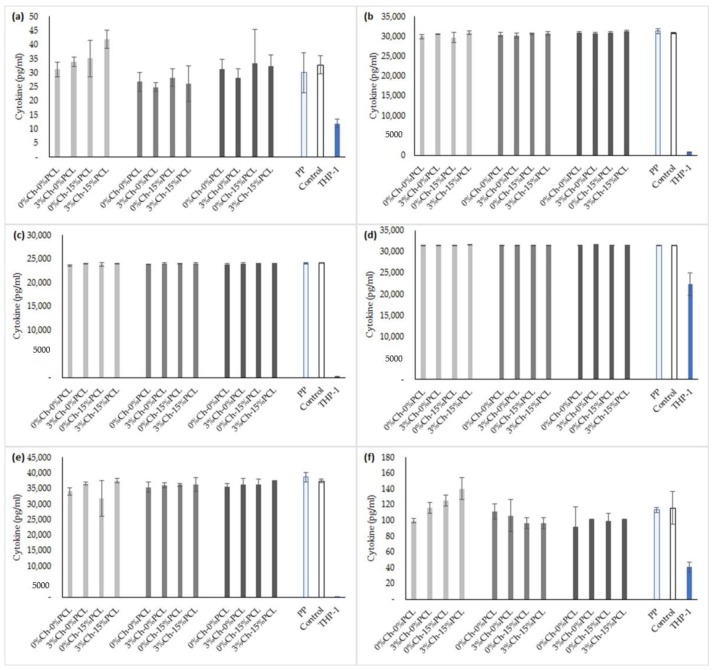
Pro-inflammatory cytokines with LPS stimulation. (**a**) INF-γ; (**b**) TNF-α; (**c**) IL-1β; (**d**) IL-8; (**e**) IL-6; (**f**) IL-2. PP: polypropylene as a reference material; Control: culture medium without PUs; THP-1: monocyte cell line. The concentration of cytokines is expressed as the mean ± SD (n = 3).

The results of the concentration of pro- and anti-inflammatory cytokines from test B are shown in Figure 17 and Figure 18, respectively. PP was tested as a reference material, and culture medium without polymer was used as a control.

As observed in the results of groups A and B, there were no significant differences between the concentrations of the cytokines expressed by the cells that were stimulated with LPS and those that were not stimulated. This may be related to the protocol that was used. A standardized protocol to differentiate THP-1 monocytes into macrophages with PMA is not yet available. Therefore, a wide variation of PMA concentrations is found—between 6 and 600 nM. The time of stimulation with PMA can vary from 3 to 72 h, and the recovery periods vary between 0 and 10 days [25,68]. It is probable that high concentrations of PMA (higher than 100 ng mL^−1^) increase the levels of expression of genes associated with inflammation and cause an increase in the secretion of pro-inflammatory cytokines, such as TNF or IL-8 [25].

As stated by Park et al. (2007), high concentrations of PMA can induce the expression of some genes during the differentiation process, which can mask the effect of post-differentiation stimuli [69]. The above results indicate that the concentrations of PMA used during the differentiation process of THP-1 monocytes into macrophages triggered the release of pro- and anti-inflammatory cytokines, and this process was prior to exposing the cells to LPS stimulation.

The results obtained for group A (Figure 15 and Figure 16) show that the PUs do not produce an inflammatory process in THP-1 macrophages. This is based on the control results (medium without PU) and the PP reference material, which showed higher values (although not always significant) in the concentrations of the evaluated pro- and anti-inflammatory cytokines.

An acute inflammation in an organism occurs due to a protective response against tissue damage caused by infectious or foreign agents. The first cytokines formed in response to bacterial lipopolysaccharides, tissue injury or infection are TNF-α and IL-1β, which act directly on specific receptors to trigger a cascade of other effectors, such as cytokines and chemokines, among others [23,25,70]. TNF-α and IL-1β have a synergic effect on inflammation, also promoted by IFN-γ through the increase of TNF-α [70]. Tumor necrosis factor (TNF-α) is a pro-inflammatory cytokine that is stimulated early in the inflammatory response [23].

One cytokine evaluated was interleukin-4 (IL-4), characterized by having a potent anti-inflammatory activity and the capacity to inhibit the synthesis of pro-inflammatory cytokines [71]. It acts on activated macrophages to reduce the effects of cytokines IL-1, TNF-α, IL-6, and IL-8 [23]. Therefore, by expressing a pro- or anti-inflammatory cytokine, the expression of its opposite is inhibited. Figure 17 shows IL-4 values near 5 pg mL^−1^, and its opposites such as TNF-α, IL-6, and IL-8 show values over 20,000 pg mL^−1^, as observed in Figure 15. Interleukin-10 (IL-10), which exerts anti-inflammatory actions on monocytes or macrophages, was also evaluated [71]. It inhibits the pro-inflammatory cytokines IL-1, TNF-α, and IL-6, stimulating the endogenous production of anti-inflammatory cytokines [23].

TNF-α plays an important role in the inflammatory response of an organism, as previously mentioned. Upon LPS stimulation, a systemic inflammatory state occurs that is characterized by increased levels of the pro-inflammatory cytokines TNF-α and IL-1β, and decreased levels of anti-inflammatory cytokines, such as IL-10 [26]. This behavior was observed in the values obtained from group B because in Figure 18a, the values obtained for TNF-α showed concentrations near 30,000 pg mL^−1^ and IL-1β had values of 25,000 pg mL^−1^, but for IL-10, mean values of 80 pg mL^−1^ were found.

Other evaluated cytokines were interleukins 2 and 6. Interleukin-2 (IL-2) is a pro-inflammatory cytokine characterized by the generation and propagation of the antigen-specific immune response [23]. Interleukin-6 (IL-6) is a cytokine with pro-inflammatory properties, and high levels are associated with severity in septic processes. IL-6 is sometimes considered an anti-inflammatory cytokine because it can induce beneficial proteins in septic shock [71].

For the two groups (A and B), the cytokines of the THP-1 monocytes that were not transformed to macrophages were evaluated to observe the basal expression levels of each cytokine. According to Chanput et al., the basal levels of cytokines of THP-1 monocytes and macrophages have values near 20 and 30 pg mL^−1^ [22]. For the study performed with PUs, cytokines IFN GAMA, IL-2, IL-5, and IL-6 had values that were within the range mentioned by the study of Chanput et al. for THP-1 monocytes, as observed in Figure 15, Figure 16, Figure 17 and Figure 18. After the differentiation of monocytes into macrophages, it was observed (Figure 17 and Figure 18) that the expression levels of all cytokines increased compared to the basal levels of the monocytes. Anti-inflammatory cytokines IL-4, IL-5, and IL-10 had increases of 55, 65, and 35%, respectively. Pro-inflammatory cytokines IL-2, IL-6, IL-8, IL-1β, IFN-γ, and TNF-α had increases of 65, 122, 29, 100, 70, and 97%, respectively. Regarding the results of the macrophages stimulated with LPS, the percent increases in the concentrations of the cytokines were similar to these.

According to the results from the reference biomaterial (PP) and the medium without PU, the behavior does not show statistically significant differences of the PUs evaluated in group B. Therefore, it can be inferred that the PUs do not have anti-inflammatory activity.

The results show that, for the differentiation of THP-1 monocytes into macrophages, in vitro standardization and optimization is necessary to achieve adequate cytokine expression results. THP-1 monocytes and macrophages could be an adequate and reliable model to evaluate the inflammatory response before conducting a more detailed study with human-derived cells [22].

## 3. Materials and Methods

### 3.1. Reagents

Castor (*Ricinus communis*) oil was purchased from Químicos Campota and Co. Ltd. (Bogotá, Colombia). Isophorone diisocyanate (IPDI), polycaprolactone diol (PCL) (average molecular weight of 2000 g mol^−1^), low molecular weight chitosan (Ch) (with a percentage of deacetylation between 75–85%), *n*-octane, 0.1 M hydrochloric acid (HCl), 0.1 M NaOH, porcine liver esterase (18 units mg^−1^), 4′,6-diamidino-2-phenylindole dihydrochloride (DAPI), and phorbol 12-myristate-13-acetate (PMA) were acquired from Sigma-Aldrich Chemical Co. (St. Louis, MO, USA). Pentaerythritol was from Merck KGaA (Darmstadt, Germany). Phosphate-buffered saline (PBS: Dulbecco’s phosphate-buffered saline), 3-(4,5-dimethyl-2-thiazolyl)-2,5-diphenyl-2H-tetrazolium bromide (MTT), 2.5% trypsin (10×), penicillin-streptomycin (10,000 units of penicillin and 10,000 μg of streptomycin per milliliter), Dulbecco’s modified Eagle medium (DMEM, 1×), Roswell Park Memorial Institute (RPMI) 1640, Live/dead^®^ Viability/Cytotoxicity Kit, Alexa Flour™ 488 phalloidin, and lipopolysaccharide (LPS, *Escherichia coli* 026:B6) were obtained from Gibco/Invitrogen (Paisley, UK). Fetal bovine serum (FBS) was from Eurobio (Les Ulis, France).

### 3.2. Biological Material

Human acute monocytic leukemia cell line, THP-1 (ATCC^®^ TIB-202™). Mouse subcutaneous connective tissue fibroblasts L929 (ATCC^®^ CCL-1). Human lung fibroblasts MRC-5 (ATCC^®^ CCL-171™). Adult human dermal fibroblast HDFa (ATCC^®^ PCS-201-012™). All biological materials were acquired from the strain library of the Universidad de La Sabana (Chía, Colombia).

### 3.3. Obtaining Polyols

The polyols were derived from castor oil (P.1, P.2, and P.3). P.1 corresponded to commercial unmodified castor oil. Polyols P.2 and P.3 were obtained by transesterification with pentaerythritol [72]. For the reaction, the castor oil temperature was raised to 120 °C for 10 min. The temperature was then further increased to 210 °C, adding pentaerythritol (1.32% and 2.64% mol pentaerythritol/mol castor oil for P.2 and P.3, respectively) and 0.05% lead oxide as a catalyst. The mixture was maintained at 210 °C for 2 h. After the reaction time, the catalyst was removed by decantation for 24 h and filtration. The hydroxyl number of the polyols were determined according to ASTM D1957-86 [73].

### 3.4. Synthesis of Polyurethanes

The PUs were synthesized by the pre-polymer method. The polyol was brought to 60 °C, and the diisocyanate was added the reactor at a constant NCO:OH ratio (1:1) [74], and maintained at 300 rpm for 5 min. Them the PCL (15% *w*/*w* of oil polyol weight) and Ch (3% *w*/*w* of oil polyol weight) were then added and maintained at 300 rpm for 5 min additional. The formation of the PU sheets was carried out by pouring the prepolymer into a steel mold. Twelve PU matrices were synthesized in sheets (15 cm × 9 cm × 0.3 cm (length × width × height)). Curing was performed at 110 °C for 12 h [72]. The synthesized PUs are identified with the following nomenclature: Pn-xCh-yPCL, where n represents the polyol used (1 for unmodified castor oil, 2 for castor oil with 1.32% pentaerythritol/mol, and 3 for the castor oil with 2.64% pentaerythritol/mol), and x and y represent the percentage of Ch and PCL, respectively.

### 3.5. Mechanical Tests

A universal testing machine EZ-LX (Shimadzu, Kyoto, Japan) was used to determine maximum stress, percent elongation, and Young’s modulus of the polyurethanes (following the ASTM D638-10 standard). A load cell of 5 kN with a crosshead speed of 25 mm min^−1^ was used [75,76]. Three samples of 40 mm × 6 mm × 3 mm (length × width × thickness) were tested.

### 3.6. Fourier-Transform Infrared Spectroscopy (FTIR)

Chemical structures were evaluated by an ATR-FTIR spectrometer (Bruker Alpha, Billerica, MA, USA), in the range from 400 to 4000 cm^−1^. The spectrum corresponds to the average of 24 scans at a spectral resolution of 4 cm^−1^ [40,77].

### 3.7. Thermal Analysis

#### 3.7.1. Thermogravimetric Analysis

Thermal behavior was evaluated in a TGA/DCS1 thermogravimetric analyzer coupled to DSC (Mettler-Toledo Inc., Schwerzenbach, Switzerland). According to ASTM D6370, the conditions used were as follows: heating speed of 25 °C min^−1^, a temperature range of 25–600 °C, nitrogen atmosphere, and samples of 15 ± 2 mg [48].

#### 3.7.2. Differential Scanning Calorimetry

Glass transition temperatures were determined by a DSC 3+ analyzer (Mettler-Toledo, Columbus, OH, USA). The conditions were as follows: temperature range from −70 °C to 150 °C, nitrogen atmosphere with 20 mL min^−1^ flow, and sample weights of 10 ± 2 mg [78].

### 3.8. Hydrophilic Character

#### 3.8.1. Contact Angle

A “Drop Shape Analysis—DSA” device (GH11, Krüss, Hamburg, Germany) was used to measure the contact angle. According to ASTM-D7334-08 (2013), it was measured with the sessile drop method using 10 μL of distilled water at 20 °C [79]. Ten measurements of each material were performed.

#### 3.8.2. Water Absorption Rate

To remove uncross-linked chains and monomer residues after synthesis, the PU materials were washed with ethanol for two days, renewing the ethanol every day. The solvent was then replaced by deionized water for two more days. The materials were dried in a vacuum chamber (0.01 mm Hg) at 37 °C for 24 h [54]. The rate of water absorption was determined by immersing the sample in distilled water until constant weight. The residual water was then removed from the samples with dry filter paper, and the samples were weighed [80]. All tests were performed in triplicate. The rate of water absorption at equilibrium was calculated by comparing the mass of the sample (m_t_) after obtaining constant weight with the initial mass (m_i_) of the sample using Equation (1):

(1) $$\%\;\mathrm{Water}\;\mathrm{absortion}\;\mathrm{of}\;\mathrm{PUs}=\left(m_{i}-m_{t}\right)/m_{i}$$

### 3.9. Determination of Density

The material density was determined using the Archimedes immersion technique using a Mettler AX 205 balance (Mettler-Toledo Inc.) with a sensitivity of 0.01 mg and a Mettler ME-33360 density determination kit. Dry samples of 5 mm × 5 mm × 3 mm were used to calculate the density in triplicate. The PUs were weighed in the air (m_air_) and then immersed in n-octane with a known density (ρ_n-octane_), and the immersion weight (m_n-octane_) was obtained at 20 °C. The density was calculated by the following Equation (2) [54]:

(2) $$\mathrm{Density}\;\mathrm{of}\;\mathrm{PUs}=\left(m_{\mathrm{aire}\;}\times {\;\rho }_{n-\mathrm{octano}}\right)/\left(m_{\mathrm{aire}\;}-m_{n-\mathrm{octano}}\right)$$

### 3.10. Dynamic Mechanical Thermal Analysis (DMTA)

The effect of temperature on the mechanical properties was evaluated by a DMTA test. A DMA 8000 thermomechanical analyzer (Perkin-Elmer, Waltham, MA, USA) was used at a frequency of 1 Hz, a deformation of 0.1%, and a temperature program between −90 °C and 150 °C, with a heating rate of 5 °C min^−1^. The storage modulus and the loss factor, tanδ [38], were determined.

### 3.11. Field Emission Scanning Electron Microscopy (FESEM)

Morphological characterization of the PUs was performed with a field-emission scanning electron microscope (FESEM, ZEISS ULTRA 55 from Oxford Instruments (Abingdon, UK). The PUs were washed with ethanol for two days, renewing the ethanol every day. The solvent was then replaced by deionized water for two more days. The materials were dried in a vacuum chamber (0.01 mm Hg) at 37 °C for 24 h [54]. The samples were coated with platinum to observe the morphology of the materials with an accelerating voltage of 5 kV [81].

### 3.12. In Vitro Biodegradability Tests

Biodegradability tests were performed in independent tests in triplicate following ASTM F1635-11. To remove uncross-linked chains and monomer residues, the PUs were washed with ethanol for two days, renewing the ethanol each day. The solvent was then replaced by deionized water for two more days. The materials were dried in a vacuum chamber at 37 °C for 24 h [54]. Samples of 5 mm × 5 mm × 3 mm were placed in the biodegradation media and incubated at 37 °C for three months, except for the enzyme medium (10 units mg^−1^ solid), which was for 21 days. After this time, the samples were washed with distilled water, dried in a vacuum chamber, and weighed. The reagents used were: 0.1 M HCl medium, 0.1 M NaOH medium, and porcine liver esterase enzymatic medium. The biodegradation rate was calculated by comparing the dry weight (w_f_) of the sample after degradation during the predetermined time with the initial dry weight (w_i_) of the sample using Equation (3) [81,82]:

(3) $$\%\;\mathrm{Degradation}\;\mathrm{of}\;\mathrm{PUs}=\left(w_{i}-w_{t}\right)/w_{i}$$

### 3.13. In Vitro Cell Viability Assay by the MTT Method

L929 mouse fibroblasts and MRC-5 and HDFa human fibroblasts were cultured in DMEM supplemented with 10% FBS and 1% penicillin-streptomycin in T-75 cell culture flasks. They were grown at 37 °C and 5% CO_2_. The cell culture medium was changed every 48 h [83]. At 100% confluence, the cells were trypsinized (trypsin-EDTA) for viability analysis.

The effect of polyurethanes on cell viability was evaluated by the MTT method defined by ISO/CD 10993-5. Cells were seeded in 96-well plates at a concentration of 4.0 × 10^4^ cells per well in supplemented medium and cultured at 37 °C and 5% CO_2_ for 24 h. Subsequently, PU cylinders of 3 mm × 2 mm (diameter × height) (previously sterilized under ultraviolet UV light (260 nm) for 30 min on each side [33]) were placed in 100 μL of supplemented medium. The materials were left in contact with the polymers for 24 h at 37 °C and 5% CO_2_. The supernatant and polymers were then removed, and the MTT solution (12 mM in PBS) was added to a total volume of 100 μL and incubated for 4 h at 37 °C. The supernatant was removed, and 100 μL of dimethyl sulfoxide was added and incubated for 15 min at 37 °C. The optical density was then determined in a plate reader (Bio-Tek ELx800 Microplate Reader, Highland Park, Winooski, VT, USA) at 570 nm. A polypropylene biomaterial (PP) was used as a positive control for cell viability, and doxorubicin (DOXO) was used as a negative control. All tests were performed in triplicate. Cell viability was determined according to Equation (4):

(4) $$\%\;\mathrm{Cell}\;\mathrm{viability}\;\mathrm{of}\;\mathrm{PUs}={\mathrm{Abs}}_{\mathrm{sample}}/{\mathrm{Abs}}_{\mathrm{control}}$$

where Abs_sample_ corresponds to the absorbance value of the cells after treatment with the PU, and Abs_control_ corresponds to the cells without treatment.

### 3.14. Immunocytochemical Techniques

In vitro cell viability assay by the live/dead kit. Cell viability was also evaluated by the live/dead kit based on plasmatic membrane integrity and intracellular esterase activity [84]. L929 mouse fibroblasts were cultured in RPMI supplemented with 10% FBS and 1% penicillin-streptomycin in T-75 cell culture flasks at 37 °C and 5% CO_2_. The cell culture medium was changed every 48 h [83]. At 100% confluence, the cells were trypsinized (trypsin-EDTA) for viability analysis.

Prior to the assay, the PUs were washed with ethanol and water for 3 days and dried in a vacuum chamber at 37 °C for 24 h [54]. PU sheets with a diameter of 4 mm (previously sterilized under UV light (260 nm) for 30 min on each side) were placed in 96-well plates [33]. Five microliters of cells, at a concentration of 1.0 × 10^4^ cells mL^−1^, were then seeded onto each material using supplemented medium and incubated at 37 °C and 5% CO_2_ for 30 min. Next, 95 μL of supplemented medium was added and incubated for 24 h at 37 °C and 5% CO_2_ [54]. The supernatant was removed, and 2 μL of calcein AM (live cell marker) staining solution and 4 μL of ethidium homodimer (dead cell marker) were added and incubated for 20 min in the dark with stirring [84]. Fluorescence measurement was performed for calcein in a Victor 1420 Multilabel Counter spectrophotometer (Perkin-Elmer, Waltham, MA, USA) at 485 nm to determine the concentration of living cells. PS was used as a positive control and latex as a negative control. All tests were performed in triplicate.

Fixation and morphological analysis with phalloidin and DAPI. L929 fibroblasts in contact with the material after 24 h of incubation were washed with PBS and fixed for 20 min with paraformaldehyde 4% *w*/*v* at 20 °C. They were then washed twice with PBS at 4 °C. Phalloidin (Alexa Flour™ 488 phalloidin) was added and incubated for 20 min to reveal F-actin. The samples were then washed twice with PBS. The cell nuclei were stained for 5 min with DAPI at a 1/5000 dilution in PBS, followed by washing twice with PBS. Next, 0.05% sodium azide was added. The cell structure was observed using an Eclipse 80i optical microscope (Nikon, Tokyo, Japan) equipped with a Nikon Intensilight Illuminator [54]. The images were analyzed by ImageJ software version 1.45k (Bethesda, MD, USA).

### 3.15. Differentiation into Macrophages and Inflammation Stimulation

A human acute monocytic leukemia cell line, THP-1, was used. The cells were cultured at 37 °C and 5% CO_2_ in RPMI medium supplemented with 10% FBS and 1% penicillin-streptomycin in T-75 cell culture flasks. For cell differentiation into macrophages, the cells were centrifuged at 2000 rpm for 10 min, resuspended in 3 mL of RPMI culture medium and seeded into 24-well plates [69] by adding 200 mM of PMA and incubating for 48 h [27]. The differentiated cells (macrophages) were washed twice with PBS to remove undifferentiated cells [26]. The macrophages that had been differentiated with PMA were stimulated with 700 ng mL^−1^ of LPS for 3 h [22].

### 3.16. Evaluation of Inflammatory Processes

With the THP-1 cell line and PUs, two independent tests were performed to evaluate the inflammatory processes of the materials in contact with the cells. The first test (group A) consisted in the direct contact of PUs on the cells differentiated into macrophages with PMA, for 24 h, to determine whether the PUs caused inflammation after contact with the cell line. The next test group (group B) consisted of PUs in contact for 24 h with macrophages differentiated with PMA and stimulated with LPS to determine whether the materials had anti-inflammatory activity.

### 3.17. Immunoassay

The MILLIPLEX^®^ MAP kit for flow cytometry was used to evaluate cytokine production (IFN-γ, IL-1β, IL-2, IL-4, IL-5, IL-6, IL-8, IL-10, and TNF-α) and release to the supernatant of the macrophages cultured in contact with PUs for 24 h, following the instructions provided by the manufacturers. A mixture of beads with different fluorescence intensities, coated with antibodies for the mentioned cytokines, was pre-incubated in the dark for one hour on an orbital shaker. Concentration standards of 3.2, 16, 80, 400, 2000 and 10,000 pg mL^−1^ were used to determine the concentration curve of each analyte. In a 96-well plate, 200 μL of wash buffer was added, the plate was shaken for 10 min, and the supernatant was discarded. Twenty-five microliters of the standards, 25 μL of running buffer, 25 μL of the stock solution, 25 μL of supernatant from cells in the appropriate wells, and 25 μL of the bead mixture were added. The plate was incubated overnight with shaking at 4 °C. The supernatant was then removed, and the samples were washed twice with 200 μL of wash buffer. Next, 25 μL of antibody was added for detection, and the samples were incubated for 1 h in the dark and with shaking. Twenty-five microliters of the streptavidin phycoerythrin binding protein was then added and incubated for 30 min with shaking in the dark. The supernatant was discarded, and the wells were washed twice with 200 μL of wash buffer. One hundred and fifty microliters of the coating liquid were then added, and the plate was read on a MAGPIX flow cytometer from MILLIPLEX MAP (Darmstadt, Germany). The results of the samples were analyzed using the xPONENT MAGPIX software (Madison, WI, USA).

### 3.18. Statistical Analysis

The results were expressed as mean values ± standard deviation (SD). The data were analyzed by means of an analysis of variance (ANOVA) and the significant differences were determined for *p* < 0.05. For the comparison between samples the *t*-Student test was used with the SPSS Statistics Software v.23 (IBM, Armonk, NY, USA).

## 4. Conclusions

PUs synthesized with castor oil polyols, isophorone diisocyanate, 15% *w*/*w* polycaprolactone and 3% *w*/*w* chitosan were used to evaluate their mechanical, physicochemical, morphological, biodegradability, and biocompatibility characteristics, along with their possible inflammatory effects. The type of polyol and additive showed a significant impact on the maximum stress, percent elongation, and contact angle of the material. Chemical modification of the polyols improves the evaluated properties due to the increased cross-linking of the resulting materials. The resulting mechanical properties of the PUs verified their dependence on the presence of hydrogen bonds, the number of hydroxyl groups in the polyols, and the interactions between the hard and soft segments of the matrix. The polyol with the highest hydroxyl index showed the highest values for the mechanical and thermal properties. The percent elongation, with a maximum of 265%, can be used to obtain resistant materials with flexibility, which allows designing biomaterials that do not cause injuries to soft tissues. The degradation rates under the study conditions showed values of less than 4%, so it can be inferred that cross-linking of the synthesized PUs hinders the degradation process. In vitro cell viability was determined using L929 mouse fibroblasts, human fibroblasts (MRC-5) and adult human dermal fibroblast (HDFa). The cell viability results of the PUs in contact with the three cell lines demonstrated that there was no effect on cell viability; therefore, it is likely that these materials could be used for direct contact with the skin without causing damage to surrounding cells. Cell viability was also determined by the live/dead kit for the L929 mouse fibroblasts. Due to the biocompatible properties of the PUs evaluated in this study, it can be inferred that they are suitable for use in biomedical applications as materials for non-absorbable biomedical sutures.

## Figures and Tables

**Table 1:** DSC thermograms of the synthesized PUs depending on the polyol used.

Polymeric Material	*T_g_* (°C)		
	P.1	P.2	P.3
0%Ch-0%PCL	−14.8	−12.8	14.8
3%Ch-0%PCL	−13.3	−1.0	13.7
0%Ch-15%PCL	−25.3	−10.2	4.7
3%Ch-15%PCL	−25.4	−14.8	2.8
